# Impact of intestinal microenvironments in obesity and bariatric surgery on shaping macrophages

**DOI:** 10.1097/IN9.0000000000000033

**Published:** 2023-11-28

**Authors:** Michael Leyderman, Joel R. Wilmore, Timothy Shope, Robert N. Cooney, Norifumi Urao

**Affiliations:** 1 Department of Pharmacology, State University of New York Upstate Medical University, Syracuse, NY, USA; 2 Department of Microbiology and Immunology, State University of New York Upstate Medical University, Syracuse, NY, USA; 3 Sepsis Interdisciplinary Research Center, State University of New York Upstate Medical University, Syracuse, NY, USA; 4 Department of Surgery, State University of New York Upstate Medical University, Syracuse, NY, USA

**Keywords:** macrophages, gastrointestinal environment, metabolism, bariatric surgery, bile acids, IgA

## Abstract

Obesity is associated with alterations in tissue composition, systemic cellular metabolism, and low-grade chronic inflammation. Macrophages are heterogenous innate immune cells ubiquitously localized throughout the body and are key components of tissue homeostasis, inflammation, wound healing, and various disease states. Macrophages are highly plastic and can switch their phenotypic polarization and change function in response to their local environments. Here, we discuss how obesity alters the intestinal microenvironment and potential key factors that can influence intestinal macrophages as well as macrophages in other organs, including adipose tissue and hematopoietic organs. As bariatric surgery can induce metabolic adaptation systemically, we discuss the potential mechanisms through which bariatric surgery reshapes macrophages in obesity.

## 1. Introduction

Over one-third of US adults suffer from obesity—a condition caused by an excessive amount of body fat with a substantially higher body mass index (BMI) ^[1]^. Obesity is a heterogeneous syndrome that has sex differences in fat distribution ^[2,3]^ and a range of severity (such as morbid obesity) ^[2]^. In general, visceral obesity is associated with multiple comorbidities, including cardiovascular diseases, obstructive sleep apnea, nonalcoholic fatty liver disease, osteoarthritis, type 2 diabetes mellitus (T2DM), and cancer ^[4]^. In contrast, subcutaneous adipose tissue is generally considered as a healthy fat depot based on population studies and fat tissue removal/transplantation data in humans and rodents ^[5,6]^. Metabolic syndrome, a cluster of conditions that increase cardiovascular risk, often involves visceral obesity along with poor metabolic health (hyperlipidemia and insulin resistance) and hypertension ^[7]^. A recent population study showed that waist-to-hip ratio had the strongest and most consistent association with mortality, supporting the idea that adiposity distribution rather than mass impacts disease risk ^[8]^.

Aberrant immune cell functions drive the development of the comorbidities of obesity ^[9]^. Efforts in the past decades have revealed the involvement of macrophage dysfunctions in this scenario ^[10,11]^. Obesity results in both an increase in tissue-localized macrophages and proinflammatory polarized macrophages such as adipose tissue macrophages (ATMs), which lead to a chronic inflammatory state in adipose tissue ^[12,13]^. Macrophages are an extremely plastic cell population and adapt to their local environment to shape a polarization phenotype and perform functions more specific to the localized tissue. Thus, local tissue environments imprint on macrophage identities as the local cellular environment produces signals that can activate transcription, resulting in a heterogeneous mix of diversified and functionally specialized macrophage subsets—in different organs as well as microanatomical localization ^[14,15]^. In obesity and its related metabolic conditions, such as insulin resistance and T2DM, recent studies have provided us with evidence of a distinct gut microbiota that is different from the nonobese gut. This dysbiosis plays a pivotal role in the changes of the downstream tissue environment from the gut to other organs ^[16,17]^ through compromised gut-barrier functions, establishing systemic inflammatory milieus such as obese adipose tissue.

Given that the gut environment can be modified by diets and drugs through the oral route, the mechanism by which the gut environment regulates intestinal macrophages and influences the macrophages of other organs or tissue compartments is of interest to discover therapeutic biomarkers for macrophage-driven inflammation under obesity. Our goal is to review the current understanding of obese tissue environments with a focus on the link between the gut environment and macrophages in the gut and other organs that may perpetuate low-grade inflammation and contribute to increased health deterioration. Bariatric surgery is associated with a reduction in comorbidities and all-cause mortality and can lead to an overall improvement in the quality of life for obese individuals ^[18]^. As anatomical rearrangement by bariatric surgery induces metabolic rewiring beyond the gut environment ^[19]^, we also explore how bariatric surgery interferes with obesity-altered gut environments and induces systemic inflammation through influencing macrophages.

## 2. Obesity alters the gut microbiome

The microbiome of a normal gut consists of more than 90% phyla *Firmicutes* and *Bacteroidetes* ^[20,21]^, anaerobic gram-negative bacteria that provide essential capacities for fermentation of nondigestible fibers, nutrient metabolism, immunomodulation, pathogen protection, and maintenance of the structural integrity of the gut mucosal barrier ^[22]^. The plasticity of the normal intestinal microbiota allows the maintenance of homeostatic equilibrium amidst different environmental changes, making sure that the metabolic and immunologic components of the microbiome do not produce drastic changes ^[23]^. The ratio between the two phyla (the *Firmicutes/Bacteroidetes* or F/B ratio) has been associated with healthy gastrointestinal tract function and tissue homeostasis in both mice ^[24]^ and humans ^[25]^. Variation of the F/B ratio is associated with various pathologies, such as an increased F/B ratio in obesity and a decreased F/B ratio in inflammatory bowel disease (IBD) ^[26]^. Its numerical values vary among studies (due to target populations and methodology), and some controversies still exist ^[26,27]^.

Obesity, as well as certain diet patterns, have been shown to disrupt the normal gut flora ^[28]^. Indeed, the link between the abnormal composition of the gut microbiota (dysbiosis) and the development of obesity has been shown in multiple studies of animals and humans. For example, a diet high in carbohydrates and fat, which predisposes to obesity, results in a 50% decrease in *Bacteroidetes* levels and an overall increase in *Firmicutes* levels ^[25]^. These changes were reversed with a change to a fat-restricted or carbohydrate-restricted diet ^[25,29]^. A decrease in biodiversity and changes in the F/B ratio (a decrease in *Bacteroidetes* and an increase in both *Firmicutes* and *Proteobacteria*) are considered strong biomarkers for obesity, as seen in both mouse and human samples ^[20,26,30]^. This has been seen in other disorders, such as IBD ^[31]^ and type 1 diabetes mellitus ^[32]^. In general, obesity-associated microbiota can be characterized by an increase in *Actinobacteria* and *Firmicutes*, a decline in *Bacteroidetes*, and diminished microbial richness and diversity ^[33]^. A systemic review and meta-analysis of gut microbiome composition in obese and nonobese subjects generally support significantly higher *Firmicutes* in obese adults, despite some controversies across studies ^[34,35]^.

Whether the diet itself or the state of obesity influences the gut microbiome is a topic of debate. One study conducted by Hildebrandt et al ^[36]^ suggested a diet-based origin for gut microbiome variabilities rather than the obese state. They demonstrated a change in gut microbiome independent of obesity by knocking out resistin-like molecule beta (RELMβ) in mice, a colonic goblet cell-specific gene whose expression is heavily dependent on the gut microbiome, and subjugated the mice to a high-fat diet (HFD), which normally induces RELMβ expression ^[36]^. In humans as well, diet induces acute alteration in the composition of the gut microbiome, suggesting that nutrients in the gut influence the homeostasis of the microbiome, which can be changed by the state of obesity ^[37]^. Interestingly, in obese mice, high-sugar intake contributes the most to microbiota disruption ^[38]^. On the other hand, *ob/ob* and/or *db/db* mice show alterations in the microbiome, including a higher F/B ratio compared with the control mice ^[39,40]^, suggesting that nondiet components can initiate the disruption of the microbiome in obesity, while increased food consumption due to leptin deficiency is likely a driver of the microbiome change ^[41]^. The pathway through bile acids (BA) or IgA discussed below could induce nondiet-related microbiome changes.

The causal relationship between an HFD or dysbiosis and health conditions has also been shown through fecal transplantation studies. Fecal microbiome transfer was shown to increase gut microbial diversity and reduce the rate of metabolic syndrome ^[42]^. Metabolic syndrome raises the risk of cardiovascular disease via atherosclerotic plaques, insulin resistance, T2DM, and cerebrovascular accidents and is improved after bariatric surgery as well ^[43,44]^. Infusion of intestinal microbiota from lean donors to male subjects with metabolic syndrome also increased insulin sensitivity and levels of bacteria that produce butyrate, a vital signaling molecule with a metabolic protective role in the duodenum and colon ^[45–47]^. *Fusimonas intestini*, which is increased in the gut of humans and mice with obesity and hyperglycemia, promotes diet-induced obesity through the production of long-chain fatty acids such as elaidate, resulting in impaired gut integrity and metabolic endotoxemia ^[48]^.

The change in gut microbiota can influence other organs as well. The whole picture of interactions between gut microbiota and circulating host microRNAs (miRNAs) in obese patients remains unclear. Four bacterial species that act as obesity biomarkers—*Dorea longicatena, Banesiela intestinihominis, Bacteroides eggerthii,* and *Haemophilus parainfluenzae*—were shown to have crosstalk between certain host miRNAs. The association leads to variable obesity-specific pathways that affect lipid turnover, fatty acid degradation, carbohydrate digestion and absorption, and other cell signaling metabolic processes ^[49]^. The alterations of the gut microbiota and subsequent changes in the intestinal structure cause metabolic stress that affects the bone marrow niche and osteoblast-adipocyte homeostasis ^[50]^.

## 3. Obesity increases epithelial permeability of the gut

In normal conditions, the translocation of microbial components across the mucosa is restricted. Both HFD and gut dysbiosis can enhance gut epithelial permeability and weaken the organization of the intestinal structure through various mechanisms, including a decrease in expression of tight junction proteins and mucin synthesis genes, abnormal architecture of the villi and crypts, including decrease in villi length, and an increase in cell death and proliferation ^[51,52]^. The resulting leaky gut of obese individuals allows for the translocation of luminal antigens into the systemic circulation and has been postulated as an important pathogenic factor in obesity and T2DM ^[53,54]^. An HFD can also stimulate a shift to barrier-disrupting hydrophobic BA as well as induce oxidative stress and apoptosis on intestinal epithelial cells. Gut microbiome dysbiosis can cause derangement of intestinal cellular turnover homeostasis, with the possibility of a gut microflora enriched in a barrier-disrupting species; this dysfunctional intestinal barrier holds a thinner mucus layer, which can lead to penetration of opportunistic bacteria and various cytokines, immunoglobulins, and macro molecules ^[52,55]^.

## 4. Obesity alters gut tissue environment influencing macrophage functions

Intestinal macrophages, which are derived from bone marrow ^[56]^, are quite heterogeneous ^[57]^ in part because of the high adaptability of these cells to different microenvironments and their constant replenishment by blood monocytes ^[58]^. Macrophages exhibit a high degree of gene-expression specialization dependent on their proximity to the gut lumen ^[59]^. Lamina propria macrophages preferentially express a proinflammatory or M1 phenotype when compared with muscularis macrophages, which display a tissue-protective or M2 phenotype ^[59]^. Further subcategorizations of intestinal macrophages in mice and humans have been shown based on microanatomical localization and/or functions as reviewed ^[60]^. While intestinal macrophages are highly phagocytic and possess antimicrobial properties, they do not produce proinflammatory cytokines in response to inflammatory stimuli in normal conditions ^[61]^, even in lamina propria macrophages. This anergic phenotype of intestinal macrophages is important in maintaining gut homeostasis. Thus, environmental factors that downregulate an inflammatory response play an important role in this tissue-protective response by macrophages in the steady state of normal physiology ^[62]^. Perturbation of macrophages can be induced by the disruption of the normal gut environment. Here we will summarize key factors in the gut environment that influence intestinal macrophage population and function.

## 5. Short-chain fatty acids maintain homeostasis of macrophages and gut environment

The changes in the microbiome and increased epithelial permeability in the gut observed in obesity are linked with the bioavailability of macrophages of bacterial-derived components, which establishes the anergic phenotype of intestinal macrophages. Short-chain fatty acids (SCFAs) are metabolic products of the gut microbiota through anaerobic fermentation of indigestible polysaccharides such as dietary fiber ^[63]^. The three main SCFAs are acetate, propionate, and butyrate. Gut bacterium from the *Bacteroidetes* phylum produce acetate and propionate, whereas the *Firmicutes* phylum mainly produces butyrate ^[29,64]^. SCFAs maintain gut homeostasis via strengthening the gut-barrier function by promoting mucus production ^[65]^ and via immune modulation. Butyrate, which acts as an energy source for colonocytes, has been shown to increase peripheral and hepatic insulin sensitivity, decrease HbA_1c_, increase leptin production in adipose tissue, stimulate beta-oxidation, and antioxidant production by the liver, and allow for greater permeability control in the gut ^[66]^. This is accomplished via butyrate acting as a ligand for metabolite-sensing G-protein coupled receptors (GPCRs), such as GPCR41 and GPCR4, which are expressed throughout the body, including peripheral nerves, enteroendocrine cells, white adipocytes, pancreatic beta cells, intestinal epithelial cells, thymus, and myeloid dendritic cells, and other myeloid-derived immune cells ^[66]^.

SCFAs modulate the mucosal immune system. They function through a GPCR cell signaling mechanism on immune cells in the intestinal epithelium, promoting protective innate lymphoid cell expansion and interleukin-22 (IL-22) ^[67]^. SCFAs positively regulate the differentiation and expansion of regulatory T cells ^[68–70]^ and of IgA-producing lamina propria plasma cells ^[71,72]^. In intestinal macrophages, SCFAs play a major role in the maintenance of homeostatic phenotype by decreasing gene expression of proinflammatory mediators such as tumor necrosis factor alpha (TNF-α), IL-6, IL-10, and nitric oxide (NO), promoting M2-like polarization, and by promoting microbial properties via bacterial membrane diffusion to reduce intracellular pH ^[73,74]^. Among SCFAs, butyrate seems to be the most potent in terms of macrophage immunomodulation. Butyrate acts as a histone deacetylase inhibitor (HDACi), favoring histone acetylation and regulating gene expressions ^[75]^. Butyrate increases total H3 acetylation and downregulates expression of proinflammatory genes such as IL-6, IL-12, and other mediators of signal transducer and activator of transcription 6 (STAT6) pathway signaling ^[73]^ as well as enhances M2 activation genes such as *Arg1* ^[74]^, rendering hyporesponsive intestinal macrophages. Moreover, butyrate promotes microbial clearance activity via mammalian target of rapamycin (mTOR) inhibition and reduction of glycolytic capacity, associated with increased histone acetylation mediated by HDAC inhibition ^[76]^.

The idea that increased histone acetylation is primarily mediated by HDAC inhibition has been challenged ^[77]^. For example, SCFAs can induce histone hyperacetylation in colon, liver, and adipose tissue, which experience SCFA concentrations that are lower than in the gut and portal vein ^[78]^. New evidence shows butyrate and propionate activate p300 at low levels through the rapid conversion to propionyl- and butyryl-coenzyme A (CoA), which are then used by acetyltransferase p300 to catalyze auto-acylation and subsequent activation of p300. Thus, propionyl- and butyryl-CoA are the main activators of histone acetylation ^[77]^. Analysis of cytokine expression in mouse epidermal immune system suggests SCFA’s effects are cell-specific and/or environment-specific: the epidermal response to the application of SCFA on the skin surface promoted cytokine expression, whereas subcutaneous administration was inhibitory ^[79]^. This may be due to different HDACs that are targeted by SCFAs ^[79]^.

Most evidence supports the protective role of SCFAs in obesity and metabolic disease. In diet-induced obese mice, SCFA supplementation reduces body weight, improves insulin sensitivity, and reduces obesity-associated inflammation ^[80–82]^. In humans, increased gut production of butyrate correlates with improved insulin response after an oral glucose tolerance test ^[83]^. Circulating but not fecal SCFAs are related to insulin sensitivity, lipolysis, and glucagon-like peptide-1 (GLP-1) concentrations ^[84]^, while acute increases in serum SCFAs were not sufficient to increase GLP-1 ^[85]^. However, obesity is associated with high fecal SCFAs ^[86,87]^, increased gut permeability, metabolic dysregulation, and hypertension ^[88]^. This raises the possibility that their overproduction may promote obesity. Indeed, propionate increases glucagon and fatty acid-binding protein 4 production, impairing insulin action in mice and humans ^[89]^. A new population study showed that nonobese participants had significantly higher weight-adjusted fecal total and individual SCFA levels, compared with their obese counterparts, with a greater influence of epidemiological background (such as residing country) on this correlation ^[90]^.

## 6. IgA induced through antigen presenting by dendritic cells and macrophages maintains homeostatic environment in the gut

IgA is a normal gut immunoglobulin that plays a role in promoting health through regulating the composition and function of the gut microbiota in mice and humans ^[91]^. IgA generated from IgA^+^ plasma cells in the lamina propria is secreted to the gut lumen, where it plays a role in controlling the composition and geographical distribution of bacterial communities in the gastrointestinal tract ^[92]^. IgA in the gut mucosa recognizes, binds, and coats bacteria and microbial proteins in a selective and nonselective manner to facilitate and maintain gut homeostasis ^[92]^. Both T-cell-dependent and independent IgA responses are induced by commensal bacteria. IgA has been shown to promote the colonization and retention of certain species, including *Bacteroides fragilis*, in the mucus layer of the colon ^[93]^.

IgA pools are derived from plasma cells residing in the gut lamina propria. However, bone marrow IgA^+^ plasma cells and increases in serum IgA have been described as a normal response to certain commensal bacteria as well ^[94]^. The production of IgA by lamina propria plasma cells is influenced by innate immune cells. A heterogeneous population of macrophages (CX3CR1^+^) seems to be essential for the IgA response during infectious colitis by inducing T-cell-dependent IgA response within both local mucosa of the colon and mesenteric lymph nodes ^[95]^. The generation of gut-protective IgA-secreting plasma cells is maintained by dendritic cell-B cell interactions in the gut-associated lymph nodes ^[96,97]^.

HFD and obesity are correlated with a reduction in IgA^+^ immune cells as well as a reduction in secretory IgA and IgA-promoting immune mediators, exposing the gut to increased inflammation ^[98]^. Importantly, the reduction of IgA in HFD feeding is associated with decreased CX3CR1^+^ macrophages in the lamina propria and mesenteric lymph nodes along with their secreted immune mediators ^[98]^. These immune mediators, such as transforming growth factor-β1 (TGF-β1), IL-5, a proliferation-inducing ligand (APRIL), also known as tumor necrosis factor ligand superfamily member 13 (TNFSF13), and retinoic acid (RA), are linked to IgA production, so the reduction of myeloid immune compartments within the lamina propria and lymph nodes contributes to reduced IgA in obesity. Furthermore, HFD-fed IgA^−/−^ mice demonstrated IgA to be an integral component that controls intestinal and adipose tissue inflammation, intestinal permeability, microbial encroachment, and the composition of the intestinal microbiome during HFD ^[98]^. Therefore, loss of IgA may contribute to increased F/B ratios associated with obesity. Importantly, bariatric surgery and metformin, by modifying the gut microbiome, can reverse the loss of IgA in HFD-fed mice ^[98]^, highlighting a strong link between the microbiome and IgA. Given that metformin also modifies metabolic pathways by activating adenosine monophosphate-activated protein kinase (AMPK) and inhibiting mTOR, metabolic changes in the gut environment (metabolic adaptation after bariatric surgery) can alter IgA availability.

Dietary manipulations eliminating soluble fiber have demonstrated a critical role for SCFA in the secretion of IgA and maintenance of microbial homeostasis in the gut ^[71]^. SCFA acting through GPR43 promotes B cell differentiation into plasma cells via increased histone acetylation and B cell metabolism ^[72]^. In contrast, dietary cholesterol and members of the intestinal microbiome induce epithelial cells to produce oxidized cholesterol byproducts (oxysterols), which are delivered in lamina propria and lymph via chylomicrons and restrain IgA secretion in duodenum lamina propria plasma cells by reducing amino acid transporter CD98 via GPR183 ^[99]^, indicating IgA can be modulated rapidly by the gut local environment.

The IgA Fc receptor FcαRI (CD89) is expressed by myeloid cells ^[100]^. Although opsonization of antigens by IgA-CD89 is implicated in IgA nephropathy ^[101]^, CD89 expression is normally downregulated in resident intestinal macrophages ^[102]^, contributing to their tolerant phenotype. Thus, there is potential for IgA to control macrophage function in peripheral tissues. However, whether the IgA reduction in obesity contributes to the development of an obese macrophage population remains unknown.

## 7. Bile acids closely linked with gut environment act through receptors in peripheral organs and become dysregulated in obesity

Bile acids endogenously synthesized from cholesterol in the liver are released into the duodenum after conjugation with glycine or taurine in the hepatocytes. The conjugated BA are transported from the small intestine into the portal circulation and hepatocytes through the two main transporters: Na^+^/taurocholate cotransporting polypeptide (NTCP) and apical sodium-dependent bile acid transporter (ASBT) ^[103]^. The gut microbiota produces secondary BA, including deoxycholic and lithocholic acids through the deconjugation of glycine and taurine followed by further biotransformation ^[104,105]^. Bile acids control gut bacteria by preventing overgrowth and inflammation ^[106,107]^. Therefore, the composition of secondary BA can vary depending on the composition of microbiota in the host. Bile acid-derived molecules mainly activate two BA-activated receptors within the gut, G-protein coupled bile acid receptor 1 (GPBAR1 or Takeda G protein-coupled receptor 5 [TGR5]) and farnesoid X receptor (FXR); the activation could be seen in the liver as well as other peripheral organs ^[104,108]^.

TGR5 cell-surface receptor is responsive to BA as agonists, and stimulation using BA has been shown to reduce phagocytosis and proinflammatory cytokine expression, subsequently promoting an anti-inflammatory M1–M2 phenotype switching (and the reverse switch shown with *GPBAR1* gene ablation) ^[66]^. Biagioli et al ^[109]^ showed the receptor was required for the maturation of monocytes within the intestinal mucosa. Dose-dependent administration of BA, such as deoxycholic acid and lithocholic acid, results in emigrated macrophages shifting toward an M2 phenotype, which causes an increase in TGF-β and IL-10 and a decrease of TNF-α, interferon-γ (IFN-γ), IL-1β, IL-6, and CCL2 mRNAs. They also demonstrated these results using a very strong GPBAR1 agonist BAR501, which showed a robust rewiring of monocyte trafficking and a drastic decrease in intestinal inflammation ^[109,110]^. Furthermore, diet-induced obesity was shown to induce systemic change in the hypothalamic BA-TGR5 system by decreasing the amount of BA species present, which normally exhibit hormone-like effects by binding to TGR5 and inhibiting the proinflammatory response in adipose tissue ^[110]^. Diet-induced obese mice lacking TGR5 exhibit enhanced inflammation and higher macrophage numbers in the adipose tissue ^[111]^. Overall, the protective role of TGR5 in metabolic health is supported by the study using TGR5-deficient mice and TGR5 agonists ^[112]^. However, the TGR5 reduction has not been observed in human obesity and insulin resistance—TGR5 gene expression levels are rather increased in obesity, and they are reduced during weight loss ^[113]^. Thus, whether protective TGR5 expression or downstream signaling is reduced in a subcategory of metabolic diseases is of interest.

FXR is another BA receptor that belongs to the nuclear hormone receptor superfamily. FXR is highly expressed in the gut-intestinal tract and liver, while adipose, cardiac, and breast tissue have low FXR expression ^[114]^. FXR is also expressed in innate immune cells such as monocytes/macrophages ^[115,116]^. Overall, activation of FXR has anti-inflammatory and protective roles. In macrophages, signals from FXR downregulate several inflammatory cytokines, including IL-1β, inducible isoforms of NO synthase (iNOS), TNF-α, IL-6, and cyclooxygenase (COX)-1 and COX-2 ^[117,118]^, and thus promote M2-like polarization ^[115]^. Similar to TGR5, the studies with FXR knockout mice and FXR agonists support the notion that FXR regulates BA metabolism and metabolic profiles to prevent metabolic conditions such as insulin resistance and atherosclerosis ^[114]^. FXR expression in obesity is context-dependent. In the ileum, mRNA levels of FXR and its targets, Shp and Fgf19, are rather increased in obese humans ^[119]^, while FXR expression is reduced in offspring of maternal obesity in the kidney ^[120]^. Interestingly, TGR5 and FXR cooperate in regulating postprandial GLP-1 secretion from intestinal L cells, regulating glucose-stimulated insulin secretion ^[121]^.

Bile acid metabolism is altered in individuals with obesity and insulin resistance ^[122]^. Although obesity and T2DM are correlated with an increase in specific BA, such as 12α-hydroxylated BA or deoxycholic acid, the main issue lies with improper BA fluctuation before and after meals ^[122,123]^, where the expression of the two BA transporters in the liver, NTCP and ASBT, is reduced in obesity (thus, a surge of serum BA after meals is blunted in obese subjects) ^[123]^. However, greater research needs to be carried out on the specific effects of a reduction in canalicular BA transport in obese patients.

## 8. Metabolic endotoxemia can lead to systemic macrophage activation

Loss of gut mucosal integrity in obesity, insulin resistance, and T2DM can lead to an influx of bacteria-derived biomolecules in the gut mucosa and eventually into the blood circulation. As we discussed above, the altered gut microbiome (dysbiosis) and the reductions of gut-barrier protective SCFA and IgA compromise gut-barrier function, leading to a condition called metabolic endotoxemia. Other than gastrointestinal tract, oral cavity, skin surface, urinary tracts, and respiratory tracts can be the sources of microbial products ^[124]^, especially in immunocompromised diabetic patients. Lipopolysaccharides (LPS) are the most studied virulence factors that are integral to the cell wall of gram-negative bacteria. The translocation of LPS can trigger a chronic low-grade inflammatory state. A low but chronic circulation of LPS, defined as circulating levels of LPS of >20 ng/mL ^[124]^, leads to metabolic endotoxemia in patients with obesity ^[36,125]^ However, the obese-associated gut sees an influx of inflammatory molecules within the mucosal environment and an M2–M1 macrophage polarization switch that continues to positively feedback the inflammatory state ^[11,51]^. These include LPS, TNF-α, IL-6, and IL-1β, which result in Nos2 upregulation ^[11,126]^. LPS is a ligand for toll-like receptors (TLRs), TLR4 and TLR2. Together with other TLRs and cell-surface pattern-recognition receptors, which can be stimulated by bacterial and cellular components, metabolic endotoxemia activates proinflammatory signaling pathways in macrophages systemically, resulting in modified macrophage function such as antigen presentation, and polarizing macrophages toward an M1 phenotype ^[127]^.

## 9. *Helicobacter pylori* can induce chronic inflammation in gastric mucosa that may prime macrophages

*Helicobacter pylori* is a well-known gram-negative bacterium that triggers chronic gastric inflammation and, like obese macrophage phenotypic switches, amplifies M1 polarization of gastric macrophages ^[128]^. While the direct link between *H. pylori* infection and obesity is controversial and not clearly shown, *H. pylori* infection influences the gut microbiota through gastric mucosa damage and local chronic inflammation that eventually spreads systemically and might contribute to obesity-associated inflammation ^[33]^. Macrophages are localized at the gastric mucosa in healthy adults and are increased in *H. pylori*-infected individuals ^[129]^. *H. pylori* infection generally promotes proinflammatory cytokine gene expressions in macrophages when they are co-cultured ^[129,130]^. Interestingly, *H. pylori* can induce a trained phenotype in which LPS-induced proinflammatory cytokine production is augmented after *H. pylori* priming ^[130]^, suggesting a possibility that a component of *H. pylori* causes metabolic and epigenetic reprogramming in monocytes/macrophages. The association between *H. pylori* and obesity has been demonstrated with mixed results ^[131]^ but in a subpopulation of subjects aged less than 50 years, *H. pylori* infection showed a possible link with higher BMI ^[132]^. Whether *H. pylori* infection can induce substantial mucosal environment impact on macrophages and obesity-associated conditions remains to be studied.

## 10. Nervous system influences macrophage functions

Neuronal activity influences the gut as the network of the enteric nervous system controls the gut activity through nerves, neurons, and neurotransmitters. It has been shown that tissue-protective muscularis macrophages control the activity of enteric neurons and gastrointestinal motility ^[133]^. In addition, norepinephrine, through *β*_2_ adrenergic receptor signaling, mediates muscularis macrophage polarization upon bacterial infection, further toward a tissue protective phenotype ^[59]^. Sympathetic neuron-associated macrophages (SAMs), a macrophage population involved in norepinephrine clearance via the transporter Slc6a2, are upregulated in the sympathetic fibers of obese mice—deletion of *Slc6a2* gene rescues the thermogenic capacities and promotes fat browning in obesity ^[134]^.

The vagus nerve (VN) establishes communication between the brain and the gastrointestinal tract and plays a role in regulating obesity, T2DM, and inflammation ^[135]^. Bidirectional communication between the brain and intestine is important in regulating food ingestion, satiety, and intestinal motility. For example, consumption of HFD results in VN dysfunction accompanied by obesity, hyperglycemia, and adipose tissue inflammation in rodents and humans ^[136]^. HFD-induced VN dysfunction mediated by downregulation of peroxisome proliferator-activated receptor gamma in VN reduces thermogenesis in the fat ^[137]^. VN stimulation has been shown to increase circulating levels of GLP-1, which lowers blood glucose, suppresses appetite, slows gastric emptying, and decreases inflammation ^[138]^. The efferent VNs are part of the cholinergic anti-inflammatory, pathway which regulates inflammation via their actions at the alpha-7 nicotinic acetylcholine receptor ^[139]^. Acetylcholine released by efferent VNs inhibits macrophage activation both in vivo and in vitro using tyrosine kinase Janus kinase 2 (JAK2) and transcription factor STAT3 to selectively activate gene expression of anticytokine proteins ^[140]^, which can be achieved by VN stimulation in lung inflammation ^[139]^ and in colitis ^[141]^. VN activity augments the phagocytic activity of macrophages in the liver ^[142]^ and intestine ^[143]^. Overall, considerable evidence supports the idea that both enteric and vagus nerves have a significant influence on macrophages in the gut and remote organs.

## 11. Obesity-perturbed adipose tissue macrophages drive metabolic diseases

**Figure 1.**
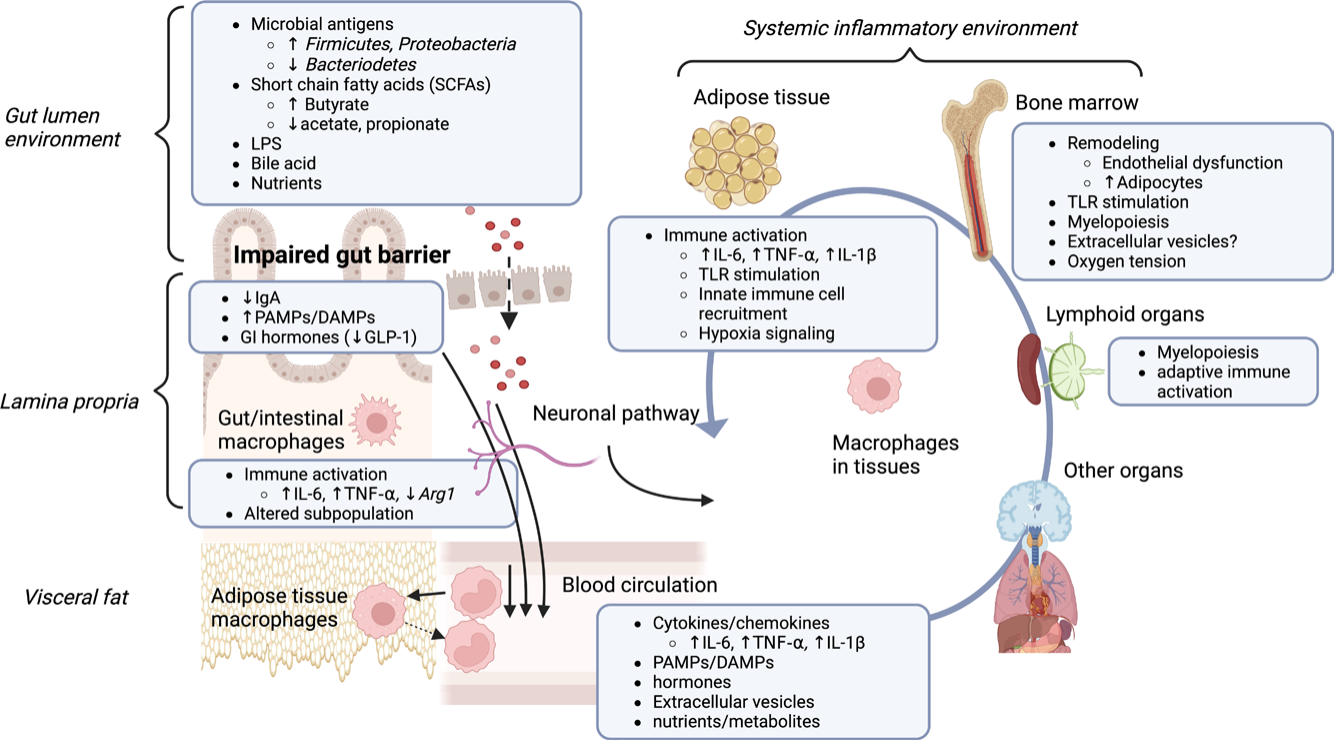
**Obesity-altered gut environment impacts on intestinal macrophages and macrophages in other tissues, sustaining a systemic inflammatory environment**. Obesity-altered gut environment impacts on intestinal macrophages and macrophages in other tissues, sustaining a systemic inflammatory environment. Homeostasis of the gut lumen environment is altered in obesity, resulting in changes in microbial antigens (upregulation of firmicutes and proteobacteria, and downregulation of bacteriodetes), which is associated with changes in short-chain fatty acids (SCFAs) (upregulation of butyrate and downregulation of acetate and propionate). These molecules as well as lipopolysaccharide (LPS) from gram-negative bacteria and bile acids can be translocated into submucosal lamina propria due to the increased gut permeability caused by the impaired gut-barrier. Nutrients through chemosensors also influence the lamina propria environment, where IgA, pattern molecules, and hormones can provide cues for immune activation and an altered subpopulation of gut/intestinal macrophages. Obesity-altered mucosal environments often result in the activation, differentiation, and polarization of immune cells and adipose tissue macrophages (ATMs) in the visceral fat. Blood circulation potentially carries the driving factors from the gut environment to the systemic environment that can influence macrophages residing in the peripheral tissues. Such factors include cytokines/chemokines (IL-6, TNF-α, and IL-1β), pathogen-associated molecular patterns (PAMPs) and damage-associated molecular patterns (DAMPs), hormones, extracellular vesicles, and nutrients/metabolites. Neuronal pathways also contribute to establishing the gut to a remote organ connection. The systemic inflammatory environment is sustained by the macrophage-driving factors in blood circulation as well as by forming inflammatory milieus in tissues. Such milieus include immune activation in the adipose tissues, remodeling, and increased myelopoiesis in the bone marrow. Lymphoid organs contribute to myelopoiesis and adaptive immune activation. As myelopoiesis influences macrophage populations through the recruitment of blood monocytes in many organs, there may be a link of obesity-altered macrophage precursors with macrophages in different compartments of the body.

Visceral fat mass is closely correlated with the gut microbiome, even in subjects with a normal BMI ^[144]^. This suggests a link between gut environment and visceral fat accumulation, which is linked with macrophage-driven inflammation ^[12,145]^. The factors in the gut environment, mucosal immune dysregulation, and subsequent changes in systemic factors drive the alteration of macrophages residing in remote organs (Figure 1).

Obese ATMs include or coexpress the genes representing M2-like macrophages such as *Arg1* ^[146,147]^, while obesity increases the genes for M1-like macrophages in adipose tissue compared with a lean control ^[147]^. Obese ATMs have been described as a mixed activated phenotype—M1-like proinflammatory cytokine production and M2-like activated phagocytic activity in mice and humans ^[148,149]^. Proteomic studies have identified a unique activation phenotype, metabolically activated obese ATMs in which independent pro- and anti-inflammatory pathways drive metabolic pathways such as lipid metabolism and glycolysis ^[150–152]^. Single-cell transcriptome analysis further confirms the unique phenotype of activated ATMs as compared with in vitro polarized M1 or M2 cultured bone marrow-derived macrophages ^[153]^. ATM subpopulations with unique signatures have been further characterized, including CD9^+^CD63^+^ lipid-associated macrophages ^[154,155]^ and interstitial perivascular phenotype ^[154,156]^. Thus, it is becoming more important to characterize macrophages based on functionality and metabolic state alongside their anatomical location to capture the heterogeneity of macrophage populations, while the M1/M2 polarization is still useful to describe an aspect of phenotypic transition associated with the development of obese ATMs and other disease-associated macrophages.

LPS signaling initiated during metabolic endotoxemia in obese individuals initiates the activation of the nuclear factor kappa-light-chain-enhancer of activated B (NF-κB), STAT1, and caspase-1 pathway, which further induces the synthesis and secretion of IL-1β along with low-grade chronic inflammation and an M1 phenotype ^[13,157]^. M1 macrophage phenotype is in part dependent on TLR4 since its deficiency drives ATMs toward an M2 phenotype ^[158]^. The NF-κB pathway mediates the induction of proinflammatory cytokines such as TNF- α, IL-1, and IL-6 ^[159]^. Upregulation of these inflammatory mediators in obesity can impair insulin action and glucose uptake in peripheral tissues ^[160]^. The induction of M1 polarization leads to greater production of the same M1-inducing molecules/cytokines from ATMs, such as TNF-α, IL-1β, IL-6, IL-12, and IL-18 ^[13,161]^. A study using chronic low-dose LPS infusion as an endotoxemia model showed that metabolic endotoxemia induced by diet increases proinflammatory cytokine in adipose tissue as well as liver and skeletal muscle in a CD14-dependent manner ^[125]^. Human data showed that a high-fat, high-sugar diet activates circulating mononuclear cells ^[162,163]^, suggesting that metabolic endotoxemia can directly or indirectly activate ATMs through the bloodstream. The expansion of adipose tissue during the development of obesity commonly results in hypertrophied adipocytes ^[164]^, which become prone to lipolysis. ATMs are accumulated through blood monocyte recruitment as well as local proliferation ^[165,166]^ during lipolysis and can work physiologically as an additional triglyceride storage source ^[167]^. Lipid-associated ATMs expressing the lipid receptor Trem2 have activated protective programs to counteract the loss of tissue-level metabolic homeostasis caused by triglyceride burden, preventing adipose tissue inflammation and adipocyte hypertrophy as well as systemic hypercholesterolemia, body fat accumulation, and glucose intolerance ^[154]^. Visceral fat has a higher rate of lipolysis than subcutaneous fat, and thus visceral fat is more prone to developing disruptive lipid metabolism that can cause a pathologic milieu in obesity as follows. Once the triglyceride burden from lipolysis overwhelms ATM’s clearing capacity and protective mechanism, saturated fatty acids (eg, palmitic acid) released via lipolysis initiate signaling mediated by pattern-recognition receptors such as TLR4 ^[168]^, further promoting inflammatory changes in macrophages ^[169]^. Altered lipid metabolism in ATMs also contributes to obese ATM development as leptin-deficient (*ob*/*ob*) mice increase the abundance of cytotoxic lipid species (eg, free cholesterol, SCFA, and saturated triglycerides) in ATMs ^[170,171]^. In addition, hypertrophic adipocytes overgrowing in diameter and moving away from the vasculature can cause adipose tissue hypoxia ^[172]^. Increased oxygen demand and consumption by adipose tissue, mediated by Ant2/Slc25a5 causing adipocyte respiration to become uncoupled ^[173]^, also contributes to adipose tissue hypoxia, triggering hypoxia-inducible factor (HIF)-1α and inflammation in adipocytes ^[174]^. HIF-1ɑ governs gene expression of proinflammatory cytokines, cellular metabolism, and angiogenesis. Thus, adipose tissue hypoxia (extracellular and intracellular) induces proinflammatory M1 polarization in a HIF-1ɑ-dependent and -independent manner in obese ATMs ^[11]^. On the other hand, using LysM-induced knockout mice, macrophage HIF-2α suppresses the expression of proinflammatory cytokines through the M2 marker, *Arg1* ^[175]^, whereas macrophage HIF-1α allows for the inflammatory microenvironment by suppressing *Arg1* ^[176]^. However, a nonhypoxia environment can activate HIF-1ɑ in ATMs through saturated fatty acids working as pseudohypoxia signals ^[176]^. Overall, local environmental cues in obese adipose tissue can drive the obese ATM phenotype.

Extracellular vesicles (EVs) released by adipocytes can have a wide range of inhibitory and stimulatory effects while playing a role in adipocyte and macrophage crosstalk. EVs contain multiple biologically active molecules, including mRNAs, long noncoding RNAs (lncRNAs), miRNAs (miRs), proteins, DNA fragments, and lipids ^[177]^. Obesity is associated with an increased level of circulating adipose tissue-derived EVs. In obese subjects, the hypertrophied adipocytes cause dysregulation of the packaging and sorting of EVs, causing increased exosome miR-802-5p content targeting heat shock protein-60 ^[178]^. miR-802 indeed suppresses endotoxin-induced macrophage activation ^[179]^ and is also implicated in insulin resistance (by acting on islet cells) ^[180]^. Similarly, EVs secreted from obese ATMs suppress insulin secretion from β cells via miR-155 ^[181]^. The exosomal miR-155 released from obese adipose tissue promotes macrophage polarization—its levels in macrophages become elevated in response to LPS, TNF-α, and IFN-β and induce M1-macrophage phenotype by activating the STAT1 pathway and inhibiting STAT6 expression ^[177,182]^. miR-223, which can be sourced from macrophages ^[183]^ and adipocytes ^[184]^, plays a role in inducing M1 to M2 formation through glycolysis alteration and repressing Krüppel-like factor 4, which leads to obesity-induced adipose inflammation ^[177,185]^. EVs released from M2-polarized macrophages ex vivo exhibit anti-inflammatory effects and resolve inflammation in HFD-fed atherosclerosis ^[186]^. Different profiles of miRNAs and lncRNAs between M1 and M2-activated macrophages ^[187]^ suggest that EVs provide signals for promoting (or inhibiting) phenotypic changes through cell-cell communication. Efforts should be made to improve our understanding of cell-cell communications and organ-organ communications through EVs in obesity.

A gut-visceral fat axis is suggested by the study using IgA-deficient obese mice, where IgA deficiency induces visceral adipose tissue inflammation with increased overall macrophage number, whereas IgA deficiency led to a reduction of homeostatic intestinal macrophages and dendritic cells ^[98]^. This indicates that ATM activity can be modified by changes in the gut environment through immune cells associated with adipose tissue. For example, adipose tissue transplantation from obese animals promotes myelopoiesis in the bone marrow, establishing a positive feedback loop between adipose tissue and bone marrow ^[188]^. In addition to bone marrow, the spleen, being a reservoir for a multitude of immune cells, supports a population of IL-10-producing B cells known as mesenteric perivascular adipose tissue (mPVAT) that help protect visceral fat tissue, specifically against HFD-induced inflammation—in splenectomized mice on a 16-week HFD, TNF-α, IL-1β, and IL-6 levels were increased, along with higher HIF-1α mRNA levels in mPVAT ^[189]^.

The egress of ATMs from adipose tissue to lymph nodes was suggested by a study on Netrin-1, which is upregulated in obesity of mice and humans and promotes retention of ATMs ^[190]^. Whether ATMs migrate beyond the regional lymph nodes is still unclear but may further establish the connection between adipose tissue and other organs. Moreover, more research needs to be conducted to determine the contribution of resident ATMs toward the inflammatory signature in adipose tissue of obese patients that comes from resident ATMs ^[165]^ compared with bone marrow-derived macrophages ^[155]^, and whether there is a crosstalk between the two ^[191]^.

## 12. Obesity-altered bone marrow may perpetuate pathological macrophage perturbations

As tissue inflammation increases the contribution of bone marrow or blood monocyte-derived macrophages to the macrophage population, obesity-induced changes in the phenotype of bone marrow-derived monocytes/macrophages and their generation from precursor cells (hematopoiesis or myelopoiesis) play integral roles in the obesity-perturbed macrophage system.

Adipokines, cytokines released from adipose tissue, serve as classic hormones that decrease insulin sensitivity and induce inflammation. They also exhibit local effects by the recruitment of inflammatory macrophages, neutrophils, and inflammatory cytokines ^[192]^. Induction of the TNF-α pathway is a prime example by which cytotoxicity and inflammatory properties of macrophages and monocytes are enhanced by both excess adipose tissue and hypertrophic adipocytes ^[192]^. Adipose tissue macrophages, by releasing inflammatory IL-1β, can promote myelopoiesis to further establish inflammation and insulin resistance in obesity ^[188]^, suggesting that the factors in adipose tissue influence bone marrow myelopoiesis, macrophage-mediated pathways, and the systemic inflammatory state. Adipokines released by excess adipose tissue have systemic effects via hypothalamic crosstalk and cause downstream changes to hunger/satiety signals ^[193–197]^.

The secretory profile of bone marrow is also impacted by obesity. As an inflammatory condition, obesity can do a wide range of damage to bone marrow macrophages. Hematopoietic stem cells and bone marrow mesenchymal cells, which lead to macrophage development via a myeloid lineage, show a decrease in secretory factors such as IL-1β, monocyte chemoattractant protein-1 (MCP-1), wingless-related integration site 10b (Wnt10b), IL-7, IL-15, and TNF-α ^[198]^. Netrin-1 protein is another factor whose expression is impaired in bone marrow cells in obesity ^[199]^ which promotes adipose tissue inflammation and insulin resistance ^[200]^ by acting as a macrophage retention signal ^[190]^.

Bone marrow consists of anatomically defined regions such as epiphysis, metaphysis, and diaphysis of the long bones, which further contains niche environments—defined anatomically as endosteal niche, periarterior niche, and perisinusoidal niche—and ones defined functionally such as hematopoietic (stem cell) niche, erythroblastic niche, and osteogenic niche. Obesity and other metabolic conditions can alter anatomically defined niches enough for the disruption of bone marrow functions that belong to those niches, as we have reviewed previously ^[201]^. As macrophages maintain and regulate some of these niches and niche functions, subsets of bone marrow resident macrophages have been characterized. These include erythroblastic island macrophages, hematopoietic stem cell niche macrophages, and osteal macrophages ^[202]^.

Obesity increases the adiposity of bone marrow ^[203,204]^. Genetic deletion of Foxc1, a transcriptional regulator of hematopoietic stem cell niche formation, led to adipocyte accumulation in the bone marrow mesenchymal cells during marrow development, and its deletion in adult marrow led to depletion of hematopoietic stem and progenitor cells with a reduction of niche-supporting chemokine CXCL12 and stem cell factor ^[205]^. Bone-resident mesenchymal stem cells that have committed adipose lineage inhibit hematopoietic regeneration, potentially by producing excessive amounts of dipeptidyl peptidase-4, a protease that is a target of diabetes therapies ^[204]^. However, bone marrow adipocytes also express a high level of stem cell factor, promoting hematopoiesis after irradiation or 5-fluorouracil (5-FU) treatment ^[206]^. These suggest bone marrow adipogenesis may impair hematopoietic niche maintenance due to both the reduction of the mesenchymal stem cell population and adipocyte-releasing factors, while some types of adipose tissue may support hematopoiesis. Transcriptomic analyses revealed that bone marrow adipose tissue is distinct from white and brown adipose tissue ^[207]^, while regional differences in the effect on hematopoiesis exist within the marrow ^[208]^. However, bone marrow adipocyte whitening leads to the proinflammatory Ly6C^high^ monocyte surge, which is associated with a glycolytic shift of monocyte metabolism, preceding the adipose tissue macrophage rise during HFD in mice ^[209]^.

Besides bone marrow resident macrophages, circulating monocytes that can home and perhaps reside in the bone marrow are impacted by obesity-induced inflammatory milieu. One explanation for M1 macrophage aggregates within inflammatory tissue could also be the high circulation of bone marrow-derived monocytes rather than resident macrophages, signaled through hypertrophic adipose tissue via MCP-1 and other inflammatory cytokines/chemokines, to move into adipose tissue and subsequently differentiate to macrophages ^[210,211]^. Furthermore, there is a causal link between the increase in local white adipose tissue in the bone marrow, and the production and activation of invasive Ly6C^high^ monocytes, a predominant tissue-infiltrating class of monocytes that is boosted in the obese phenotype and differentiates to proinflammatory M1 ATMs ^[209,212,213]^.

While most evidence has shown the link between ATMs and bone marrow hematopoiesis in obesity and other metabolic diseases, a recent study has provided evidence of the gut-bone marrow axis. The microbiota-derived butyrate, a SCFA, has been associated with bone marrow macrophage activity, in which iron recycling and iron availability for hematopoietic stem cells are regulated by red blood cell phagocytosis ^[214]^, suggesting that factors in the gut influence resident macrophage function in the remote organ. Future studies need to address the gut-bone marrow axis in obesity.

## 13. Bariatric surgery induces immunologic reshaping of macrophages and tissues

Suffice it to say that bariatric surgery has been proven to be very successful in altering the inflammatory phenotype seen in obese individuals, as chronic low-grade inflammation and its associated comorbidities of obesity are usually resolved after surgery ^[215,216]^. The landmark, Swedish Obese Subjects Study, showed bariatric surgery to be associated with a longer life expectancy than usual standard medicine treatment ^[217]^.

**Figure 2.**
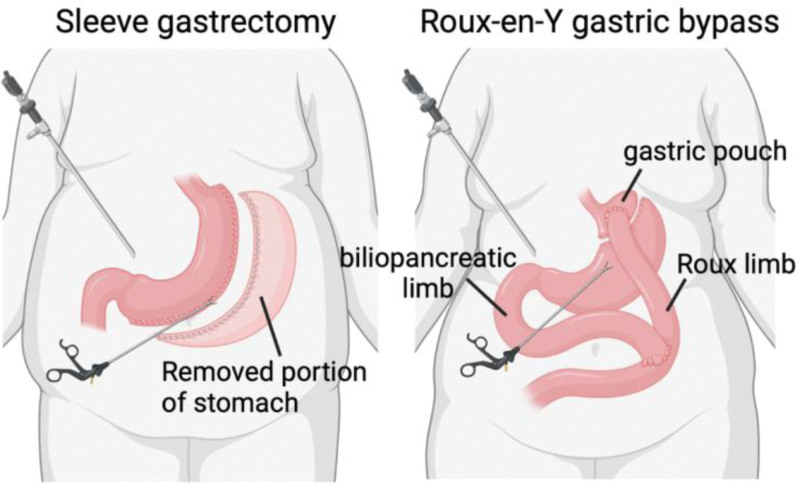
**Common bariatric surgery procedures.** The laparoscopic sleeve gastrectomy (LSG) procedure resects the greater curvature of the stomach, creating a smaller, tubular gastric viscus (Left). The Roux-en-Y gastric bypass (RYGB) procedure involves several steps: creating a small gastric pouch by stapling the proximal stomach, creating a Roux limb, performing a jejunojejunostomy connecting the biliopancreatic limb and Roux limb, and small caliber gastrojejunostomy between the proximal gastric pouch and Roux limb (Right).

The most common bariatric surgical procedures are laparoscopic sleeve gastrectomy (LSG) and laparoscopic Roux-en-Y laparoscopic gastric bypass (RYGB). The LSG procedure resects the greater curvature of the stomach creating a smaller, tubular gastric viscus (Figure 2). Although LSG was initially believed to be primarily a mechanically restrictive procedure, it also appears to have metabolic adaptation by reducing ghrelin secretion, enhancing gastric emptying, and creating changes in the BA and enteric hormones ^[217,218]^. The mechanism of LSG-reduced ghrelin, which is a hunger hormone, can be explained by the removal of ghrelin-producing gastric fundus and body of the stomach ^[219]^. A ghrelin receptor, the growth hormone secretagogue receptor (GHS-R), is expressed on human T cells and monocytes and is responsible for counterbalancing leptin-induced proinflammatory cytokine expression ^[220]^. In aged mice, GHS-R ablation promotes a phenotypical shift toward anti-inflammatory M2 in ATMs, partly through increased norepinephrine production ^[221]^. However, there is no evidence that LSG-reduced ghrelin promotes proinflammatory polarization in macrophages. Meal-stimulated BA in plasma have been correlated by increasing glycine-amidated hyocholic acid alongside greater BMI loss after LSG ^[222]^, suggesting that the BA pathway, likely through TGR5 and FXR, is involved in LSG-induced metabolic adaptation.

The RYGB procedure involves several steps: creating a small gastric pouch (about 2 ounces in size) by stapling the proximal stomach, creating a Roux limb (approximately 100–150 cm), performing a jejunojejunostomy connecting the biliopancreatic limb and Roux limb, and finally performing a small caliber gastrojejunostomy (12 mm) between the proximal gastric pouch and Roux limb (Figure 2). In RYGB, BA circuitry is aided back to normal by increasing serum BA levels and prompting greater absorption of BA back into hepatic circulation in both the ileum and the liver ^[223,224]^. Improvements in obesity-related medical problems after RYGB were initially attributed to weight loss. However, the observation that RYGB patients with T2DM demonstrate significant improvements in glycemic control shortly after surgery and before losing weight ^[225]^ challenged this concept. Early improvements in glycemic control after RYGB appear to be due in part to enhanced GLP-1 secretion, changes in intestinal glucose transport, and improvements in insulin sensitivity ^[226]^ as well as anti-inflammatory effects ^[222]^. In a rodent model of RYGB, bile diversion to the ileum improved glucose homeostasis via intestinal FXR-mediated GLP-1 production as well as alteration of the microbiota ^[227]^. Indeed, GLP-1 levels were found to be higher after RYGB in multiple human studies ^[228]^.

Bariatric surgery has been shown to reverse some of these changes that spawn from disruption of the gut microbiota ^[229–231]^. For example, RYGB increases the levels of secreted gut IgA antibodies and of total fecal IgA antibodies 6 months after surgery ^[227]^. Bariatric surgery can affect the interplay of BA and gut microbiota in a way that alleviates inflammation by increasing *Bacteroidetes* and decreasing *Firmicutes*, causing strong metabolic improvement in patients ^[232]^. In addition, regarding the location of the nerve cut, rodent models identified that RYGB, but not LSG, can trigger microglia activation in VN systems ^[233]^.

Different from weight loss by diet change, RYGB sustains metabolic improvements in the duodenum, jejunum, ileum, adipose tissue, liver, and skeletal muscles, and the effects are conserved between mice and humans ^[227]^. This study pointed out that the tissue adaptation process after bariatric surgery may be coordinated by HIF-1α activation, likely through mTOR pathway activation, which increases the utilization of systemic glucose and augments the uptake of glutamine and cholesterol ^[227]^. In a rodent model, diet-induced obesity increases HIF-2α but not HIF-1α in the intestine ^[234]^, and sleeve gastrectomy induces HIF-2α in the upper intestine ^[235]^. While the mechanism of HIF-2α upregulation by obesity and gastrectomy is not clear, the induction of HIF-1α and HIF-2α by gut-specific von Hippel-Lindau deletion improves glucose tolerance ^[235]^, whereas HIF-2α is dispensable for the metabolic improvement by gastrectomy ^[235]^. These suggest that HIF signaling in the gut is closely associated with metabolic health and HIF-1α mediates metabolic adaptation after bariatric surgery. As a potential mechanism that may lead to sustained metabolic adaptation by bariatric surgery, epigenetic changes in metabolic pathways in the intestine and other organs have also been implicated ^[226]^. The epigenetic profiles of obese patients, such as DNA methylation, were reversed after bariatric surgery ^[236]^.

Alterations in adipose levels of cytokines after bariatric surgery remain controversial, with levels of pre- and post-surgical inflammatory profiling yielding different results ^[237]^—adipose tissue mRNA levels of TNFα remain controversial, as adipocytokine and acute-phase protein levels pre- and post-surgery remain inconsistent despite the demonstrated impact on T2DM remission and weight loss ^[237,238]^. After RYGB, patients experience a significant decrease in plasma concentrations of leptin, insulin, C-reactive peptide, plasminogen activator urokinase receptor, and two acute-phase proteins, serum amyloid A and orosomucoid ^[239,240]^. The decrease in macrophages can also be attributed to the decrease in the genes of macrophage-attracting proteins such as MCP-1, PLAUR, colony-stimulating factor 3 (CSF-3), HIF-1α, and IL-6 in the adipose tissue ^[240]^. One study showed a decrease in IL-36γ in human adipocytes and macrophages after surgery, suggesting the role of IL-36γ in adipocyte tissue inflammation ^[241]^. Several studies showed a reduction of overall ATMs ^[240]^, and robust evidence remains for bariatric surgery reversing macrophage phenotypes from M1 to M2 ^[238]^. Other studies showed that postbariatric surgery, CD40^+^ cells (M1-like) decreased and CD206^+^ and CD163^+^ (M2-like) increased, with an overall significant decrease in the short-term presence of total ATMs in subcutaneous adipose tissue ^[240,242]^. Other studies showed differences in collagen remodeling ^[243]^ and angiogenesis ^[244]^, two processes that are strongly accomplished via macrophages, in adipose tissue after bariatric surgery. In humans, cytokine profiles in the blood can be reversed after bariatric surgery—elevated CCL14, soluble vascular endothelial growth factor receptor 2 (VEGFR2), and platelet-derived growth factor BB in obesity are reduced, and lower CXCL12, CCL11, and CCL27 in obesity are increased ^[245]^. C-reactive protein, IL-6, TNF-α, and other proinflammatory cytokines in the blood have also been shown to be reduced by 6 months after bariatric surgery ^[246–248]^. Overall, bariatric surgery can induce the resolution of inflammation based on cytokine profile, and this effect may be proceeded by metabolic adaptation such as insulin resistance and lipid parameters ^[249]^, implicating the involvement of the immune system such as macrophages.

## 14. Conclusion and future perspectives

We discussed the impact of an obesity-altered gut environment on macrophages, which are ubiquitous, highly plastic, and heterogeneous in homeostasis. Evidence shows the close association of the gut environment with visceral fat, bone marrow, and further systemic organs (Figure 1). Metabolome profiling identifies clinically meaningful heterogeneity of cardiovascular risks in obesity rather than BMI ^[250]^. As discussed, microbiome and gut mucosal integrity are associated with circulating factors, and how macrophages in each organ contribute to the metabolome will be a focus of macrophage biology in obesity.

Technological advancement, particularly in the methodology of genomic/epigenomic analysis at the single-cell level, allows us to demonstrate disease-associated macrophages with heterogeneity of cell populations. For example, Trem2-positive lipid-associated macrophages, in both rodents and humans, are induced by an obese environment, which prevents adipocyte hypertrophy, inflammation, and systemic metabolic dysregulation ^[154]^. Moreover, the multi-omics approach identified early priming of macrophage precursors, myeloid progenitors, in terms of differentiation fate ^[251]^. As long-term innate immune memory can be developed in macrophage precursors in the bone marrow ^[252]^ and hematopoiesis continues to regulate macrophage functions ^[201]^, investigations of the impact of hematopoiesis on macrophages in the different compartments would help to advance this research field.

The complex interplay between neuronal activity, the gut microenvironment, and macrophage functions remain to be elucidated. Norepinephrine through β_2_ adrenergic receptor signaling mediates muscularis macrophage polarization upon bacterial infection further toward a tissue protective phenotype ^[59]^, while SAMs, a macrophage population involved in norepinephrine clearance, are upregulated in sympathetic fibers of obese mice ^[134]^. Acetylcholine released by efferent VN inhibits macrophage activation through the JAK2-STAT3 pathway both in vivo and in vitro ^[139–141]^. HFD intake results in VN dysfunction accompanied by obesity, hyperglycemia, and adipose tissue inflammation in rodents and humans ^[136]^. Overall, considerable evidence supports the idea that autonomic nerves have a significant influence on macrophages in the gut and remote organs.

Finally, the long-term success of bariatric surgery as a metabolic intervention for obese patients ^[18]^ prompted us to better understand the mechanisms behind metabolic adaptation after bariatric surgery. It remains to be understood whether macrophages participate in metabolic adaptation and whether bariatric surgery can normalize the immune memory developed by obesity. Future research will address the mechanisms of perturbed macrophages in obesity, which can lead to establishing a risk-assessing biomarker and the development of a novel therapy.

## Conflicts of interest

The authors declare that there are no conflicts of interest.

## Funding

Funding support from the National Institutes of Health (NIH) National Institute of Diabetes and Digestive and Kidney Diseases (R01 DK111489 to NU, and R01 DK122332 to RNC), and National Institute of General Medical Sciences (R35GM147560 to JRW, and R01GM144624 to NU).

## Acknowledgments

We acknowledge funding support from the National Institutes of Health (NIH) National Institute of Diabetes and Digestive and Kidney Diseases (R01 DK111489 to NU, and R01 DK122332 to RNC), and National Institute of General Medical Sciences (R35GM147560 to JRW, and R01GM144624 to NU). The content of this article was written entirely by the authors listed. No ghostwriters were used to write this article. The figures were created with BioRender.com.
